# Photosynthesis and yield enhancements in rice by foliar magnesium supply under variable soil nitrogen applications

**DOI:** 10.3389/fpls.2026.1739257

**Published:** 2026-03-20

**Authors:** Houjun Liu, Yifan Wu, Yandi Jin, Miaolin Song, Haobo Jin, Jian Dai, Jinfeng Yang

**Affiliations:** National Engineering Research Center for Efficient Utilization of Soil and Fertilizer Resources, College of Land and Environment, Shenyang Agricultural University, Shenyang, China

**Keywords:** foliar spraying Mg fertilizer, N fertilizer, N and Mg interaction, photosynthesis, rice

## Abstract

**Introduction:**

Magnesium (Mg) and nitrogen (N) are both essential elements for plant growth, and their synergistic interactions in plants have been widely reported. This study aims to clarify how foliar Mg application enhances photosynthesis performance, the transport of photosynthetic products, and yield formation under variable soil nitrogen applications.

**Methods:**

Rice was cultivated in soil supplied with 0.4, 0.3, and 0.2 g kg^−1^ N as urea in a pot experiment and subjected to foliar Mg application of 0%, 2%, and 4% as MgSO_4_·7H_2_O at the jointing and booting stages.

**Results:**

The results demonstrated that foliar Mg application improved the net photosynthetic rate and soluble sugar content in leaves, and increased the starch, protein, and dry weight of grains. The increases were more pronounced under Mg_4_ (4% MgSO_4_·7H_2_O), particularly when combined with the N_0.3_ or N_0.2_ treatments. For example, the highest grain weight (151 g pot^−1^) was recorded under N_0.4_Mg_4_, 11.7% higher than N_0.4_Mg_0_, and the grain weights of N_0.3_Mg_4_ and N_0.2_Mg_4_ were 17.0% and 7.9% higher than N_0.3_Mg_0_ and N_0.2_Mg_0_, respectively. The highest starch content (759.7 g kg^−1^) was observed under the N_0.4_Mg_2_ and N_0.4_Mg_4_ treatments. Compared with Mg_0_, Mg_2_ and Mg_4_ increased starch contents by 0.44% and 0.45% under the N_0.4_ level, by 0.35% and 0.57% under the N_0.3_ level, and by 2.21% and 1.71% under the N_0.2_ level, respectively. Mg application effectively regulated the photosynthetic framework by strengthening maximal fluorescence (Fm), photosystem II (PSII), reaction center pool size (Area), quantum yield of electron transport (φEo), and the total performance index (PI_total_) for energy conservation from exciton trapping to the reduction of photosystem I.

**Discussion:**

This enhancement in photosynthetic efficiency promoted carbohydrate accumulation, thereby providing a physiological basis for improvements in grain yield and quality. Overall, our research demonstrated that N_0.4_Mg_4_ is more favorable for improving rice yield and quality, while N_0.2_Mg_4_ is more beneficial to raising nitrogen use efficiency. These findings prove the agronomic significance of foliar Mg application under variable soil nitrogen regimes for sustainable rice production.

## 1 Introduction

An adequate Mg fertilization rate can increase crop yield by an average of 8.5% worldwide and improve the quality of various agricultural products (Grzebisz, 2013; Hauer-Jakli & Trankner, 2019; Jaghdani et al., 2021; Andrzejewska et al., 2025; Wang et al., 2020; Chen et al., 2022). As an essential nutrient in plants, approximately 35% of total Mg is localized in the chloroplasts of leaf cells, where it plays a key role in chlorophyll synthesis, light harvesting, and the activation of photosynthetic enzymes (Ishfaq et al., 2022). Furthermore, Mg facilitates the source–sink transport of carbohydrates by preventing sugar accumulation in leaves and thereby reducing feedback inhibition of photosynthesis (Farhat et al., 2016). Thus, Mg makes a substantial contribution to the production and allocation of photoassimilates (Farhat et al., 2016; Tian et al., 2021). Foliar Mg application has been reported to enhance photosynthetic capacity in broad bean, increase the transcription level of ATPase in the plasma membrane of tender leaves, and ultimately increase yield (Neuhaus et al., 2013). In cereal crops such as rice, maize, and barley, Mg fertilization significantly improves grain yield and quality (Liu et al., 2021; Khokhar et al., 2022; He et al., 2024).

Nitrogen (N) is another essential nutrient and is fundamental to plant growth and agricultural production (Lee et al., 2020; Guo et al., 2021; Mu & Chen, 2021; You et al., 2023) It is indispensable for photosynthesis, as it plays a crucial role in the synthesis of photosynthetic components, including biotin carboxyl carrier proteins, which are key constituents of amino acids, nucleic acids, and chlorophyll. However, excessive N application usually causes crop overgrowth, grain filling inadequacy, and ultimately production drawdown (Chen et al., 2022). Moreover, excessive N fertilizer leads to reduced nitrogen use efficiency (NUE) and increased environmental impacts. In contrast, an appropriate amount of N fertilizer can balance rice yield and grain quality by enhancing root activity, photosynthetic capacity, nutrient absorption, and transport, as well as the accumulation and redistribution of dry matter (Zhao and Fitzgerald, 2013; Guo et al., 2022). Moderate N reduction has also been reported to enhance NUE, agronomic efficiency, and nitrogen harvest index in crops such as ramie (Saleem et al., 2022).

There is an important interaction between Mg and N in various physiological and biochemical processes within plants (Lamichhane et al., 2023; Li et al., 2023; Tian et al., 2023; Yang et al., 2024). Adequate Mg supply enhances N uptake and utilization by regulating Mg-ATP formation and the activity of enzymes involved in nitrogen assimilation (Jezek et al., 2015). Foliar Mg application promotes N accumulation in leaves and grains by improving N uptake efficiency (Zhang et al., 2020). Conversely, N availability influences Mg uptake by affecting glutamine synthetase activity (Geng et al., 2021). The combined application of Mg and N has been shown to enhance chlorophyll content, photosynthetic performance, and yield in blueberry (Pinzón-Sandoval et al., 2023), as well as to improve photosynthetic characteristics, antioxidant metabolism, and endogenous hormonal balance in Chinese cabbage (Mao et al., 2022). Research on Mg–N interactions has gradually expanded from cash crops to field crops, with increasing attention being paid to rice (Dromantienė et al., 2017; He et al., 2024).

In our previous research, we demonstrated that Mg fertilization enhanced N uptake and yield formation of rice under excessive and optimal N supply, and the yield increase was more significant under the optimal N treatment (He et al., 2024). However, limited attention has been given to the effects of Mg on the photosynthetic process, the transport of photosynthetic products, and the yield and grain quality formation under different soil N applications. In addition, the underlying mechanisms of the synergistic interaction between Mg and N regarding photosynthesis in rice remain poorly understood. We hypothesized that Mg application could enhance photosynthetic performance, thereby increasing yield and improving grain quality under varying soil N conditions, and there exists an optimal combination of Mg and N for rice production. The present study is expected to provide theoretical insights and technical guidance for the rational management of Mg and N fertilizers in rice-growing regions.

## 2 Materials and methods

### 2.1 Experiment site and plant material

The pot experiment was conducted in a greenhouse at Shenyang Agricultural University (123°57′E, 41°83′N), Liaoning Province, China. The region has a temperate monsoon climate, with an average annual temperature ranging from 7.5 °C to 8.5 °C, approximately 64% relative humidity, and high rainfall from June to August. The greenhouse was maintained at an average sunshine duration of 9–15 h and temperatures ranging from 15 °C to 30 °C. The soil used in the experiment was collected from a paddy field in Shenyang, Liaoning, China. The soil was clay loam, and its basic properties are listed in **Table 1**. Rice seedlings were transplanted on 31 May and harvested on 22 October 2023. Rice seedlings were previously cultivated as follows: the seeds were soaked in water for 48 h in a dark chamber at room temperature and then sown in well-cultivated clay loam in PVC trays. The 20-day-old seedlings were transplanted in pots. The rice variety was Shennong 9816.

**Table 1:** Basic properties of soil in experiment.

pH	Alkali-hydrolyzale N (mg·kg^−1^)	Available phosphorus (mg·kg^−1^)	Available potassium (mg·kg^−1^)	Exchangeable Mg (mg·kg^−1^)
6.45	57.08	14.73	74.64	332.26

### 2.2 Design of experiment

The experiment contained three nitrogen rates (0.4, 0.3, and 0.2 g kg^−1^, denoted as N_0.4_, N_0.3_, and N_0.2_, respectively) and three magnesium rates (0%, 2%, and 4% Mg applied as MgSO_4_·7H_2_O, corresponding to 0, 26, and 52 mg MgO kg^−1^ soil, denoted as Mg_0_, Mg_2_, and Mg_4_, respectively). Urea and magnesium sulfate heptahydrate (MgSO_4_·7H_2_O, Mg ≥ 9.7%) were selected as N and Mg fertilizers, respectively. Nine treatments were established: N_0.4_Mg_0_, N_0.4_Mg_2_, N_0.4_Mg_4_, N_0.3_Mg_0_, N_0.3_Mg_2_, N_0.3_Mg_4_, N_0.2_Mg_0_, N_0.2_Mg_2_, and N_0.2_Mg_4_. Basal fertilization comprised 40% N (urea), 100% P_2_O_5_ (potassium dihydrogen phosphate, 0.15 g kg^−1^ soil), and 50% K_2_O (potassium chloride, 0.1 g kg^−1^ soil); tillering fertilizer included 35% N and 50% K_2_O; and panicle fertilizer was the remaining 25% N. Each treatment was replicated three times, and each replicate contained four seedlings. An aqueous solution of MgSO_4_·7H_2_O was sprayed onto rice leaves at the jointing and booting stages.

### 2.3 Determination of photosynthetic parameters and chlorophyll fluorescence parameters in functional leaves

Photosynthetic and chlorophyll fluorescence parameters (as described in **Table 2**) were measured in the morning (09:00–11:00) at 5–7 days after Mg application during the jointing and booting stages of rice. Fully expanded functional leaves with high photosynthetic activity were selected, and photosynthetic parameters were measured using a portable photosynthesis system (LI-6400XT, LI-COR, USA). Subsequently, the selected leaves were dark-adapted for 30 min, and chlorophyll a fluorescence was measured using a portable PEA fluorimeter (Hansatech Instruments Ltd., UK) (Chen et al., 2021).

**Table 2:** List of fluorescence parameters.

Parameters	Explanation
Fo	Minimal fluorescence, when all PSII RCs are open
Fm	Maximal recorded fluorescence intensity, at the peak P of OJIP
Fv	Maximal variable fluorescence
Fv/Fm	The maximal photochemical efficiency of PSII
Area	PSII receptor library size
Sm	Normalized total complementary area
Mo	Approximated initial slope of the fluorescence transient
*V*_J_	Relative variable fluorescence intensity at the J-step
Ψo	Probability/efficiency (at *t* = 0) that a trapped exciton moves an electron into the electron transport chain beyond *Q*_A_
φEo	Quantum yield of electron transport (at *t* = 0)
ABS/RC	Absorption flux (of antenna chlorophylls) per RC
TRo/RC	Trapping flux (leading to QA reduction) per RC
ETo/RC	Electron transport flux (further than QA−) per RC
REo/RC	Electron flux reducing end electron acceptors at the photosystem I (PSI) acceptor side per RC
DIo/RC	Dissipated energy flux per RC (at *t* = 0)
PI_total_	Performance index (potential) for energy conservation from exciton to the reduction of PSI end acceptors

### 2.4 Collection of plant samples

Following the above measurements, these functional leaves were cut and rinsed with distilled water. A part of them was stored at −20 °C for soluble sugar measurements. Another part was dried for Mg measurements. At maturity, rice plants were harvested and separated into stems, leaves, grains, and roots. Fresh and dry weights of every part were recorded. Dried samples were ground to fine powder for determining protein, starch, and nutrient contents.2.5 Determination of soluble sugars in functional leaves.

The anthrone-ethyl acetate-sulfuric acid colorimetric method was used to quantify soluble sugars. A leaf sample was taken from a −20°C refrigerator, cut into small pieces, and boiled twice in 5 mL of water for 30 min each, then diluted to 50 mL. A 0.5-mL aliquot of the extract was mixed with 0.5 mL of anthrone-ethyl acetate reagent and 5 mL of concentrated H_2_SO_4_ and then boiled for 1 min. Soluble sugars were measured at 620 nm using a UV-visible spectrophotometer (752N, Shanghai, China) (Fan and Ruan, 2015).

### 2.6 Determination of Mg in functional leaves

The dried leaf sample was digested in a mixture of 2 mL of 30% H_2_O_2_, 5 mL of 65% HNO_3_, and 1 mL of ultrapure water in a closed microwave system (MarsExpress; CEMCorp., Matthews, NC, USA). After digestion, the solution was diluted to 50 mL with ultrapure water, and Mg concentration in solution was measured using an atomic absorption spectrometer (PinAAcle 900, Perkin Elmer, Shanghai, China) (Bao, 2000).

### 2.7 Determination of starch and protein in grains

The starch content in the grain was determined using copper sulfate reduction–titration after the sample was hydrolyzed by dilute hydrochloric acid (1% HCl) in boiled water. The protein content in the grain was analyzed as follows: the sample was digested with concentrated sulfuric acid in the presence of a catalyst (CuSO_4_ + K_2_SO_4_), and then total N in the digestion solution was measured using the Kjeldahl method to calculate protein content (Bao, 2000).

### 2.8 Statistical analysis

Data processing and visualization were performed using Microsoft Office Excel 2016 software. Two-factor analysis was conducted using SPSS 23.0 Pro statistical software. The significance of differences between groups was analyzed using the LSD method at a significance level of *p* < 0.05.

## 3 Results

### 3.1 Mg contents in functional leaves

**Figure 1:**
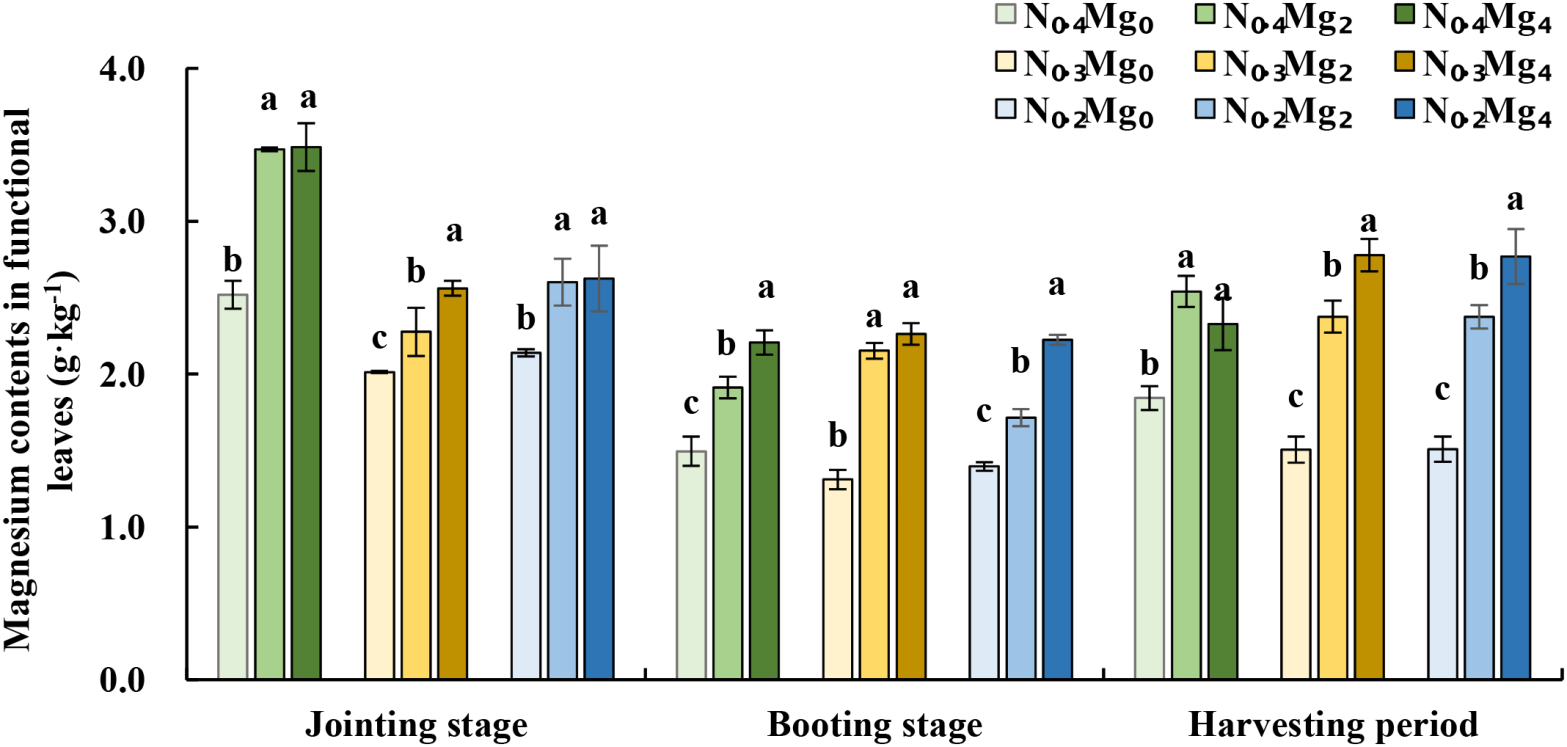
Mg contents in functional leaves at different treatments at the joining stage, booting stage, and harvest stage of rice under different Mg and N treatments. Mg treatments—Mg_0_, Mg_2_, and Mg_4_ (corresponding to 0%, 2%, and 4% MgSO_4_•7H_2_O); N treatments—N_0.4_, N_0.3_, AND N_0.2_ (corresponding to 0.4, 0.3, and 0.2 g N kg^−1^ soil). The bar letters in the same column represent significant differences between different Mg concentrations for the same N treatment at each stage. Data are means (*n* = 3).

The Mg contents in functional leaves decreased under N_0.3_ and N_0.2_ compared to N_0.4_ treatment. At the jointing stage, the Mg contents decreased by 20%–30% regardless of the Mg application rate, whereas at the booting and harvesting stages, it decreased by 6%–18% only in the absence of Mg application (**Figure 1**). At the three stages, foliar Mg application (Mg_2_ and Mg_4_) significantly increased Mg contents in functional leaves by 13%–84%. These results indicate that the synergistic interaction between Mg and N is important for Mg accumulation in functional leaves.

### 3.2 Photosynthesis parameters in leaves

At both jointing and booting stages, N_0.2_ and N_0.3_ treatments significantly decreased the net photosynthetic rate, stomatal conductance, and transpiration rate in leaves, whereas Mg_2_ and Mg_4_ application markedly improved these parameters. The effect of magnesium on photosynthesis parameters was more pronounced under N_0.2_ and N_0.3_ (**Table 3**). At the jointing stage, the highest net photosynthetic rate (21.5 μmol CO_2_ m^−2^ s^−1^) was observed under N_0.4_Mg_2_. Compared with Mg_0_, Mg_4_ increased the net photosynthetic rate by 31.5% and 13.7% under N_0.2_ and N_0.3_, respectively. At the booting stage, Mg_4_ further increased photosynthetic rate by 13.8% (N_0.2_) and 29.4% (N_0.3_). Under N_0.4_, Mg application reduced stomatal conductance and transpiration rate, whereas under N_0.2_, it significantly enhanced both parameters. In contrast, intercellular CO_2_ concentration did not differ significantly among all treatments.

**Table 3:** Photosynthesis parameters in leaves under different treatments at joining and booting stages of rice under different Mg and N treatments.

Treatment	Net photosynthetic rate (μmol CO_2_ m^−2^ s^−1^)		Stomatal conductance (mol H_2_O m^−2^ s^−1^)		Intercellular CO_2_ concentration (μmol CO_2_ mol^-1^)		Transpiration rate (mmol H_2_O m^−2^ s^−1^)	
	Jointing stage	Booting stage	Jointing stage	Booting stage	Jointing stage	Booting stage	Jointing stage	Booting stage
N_0.4_Mg_0_	18.8 ± 0.83 b	17.3 ± 0.39 a	0.507 ± 0.008 a	0.267 ± 0.005 a	287 ± 10.6 a	246 ± 14.5 a	7.9 ± 0.67 a	8.9 ± 0.45 a
N_0.4_Mg_2_	21.5 ± 0.27 a	16.8 ± 1.45 a	0.487 ± 0.002 ab	0.259 ± 0.005 a	285 ± 1.25 a	237 ± 10.1 a	7.8 ± 0.65 a	8.3 ± 0.15 ab
N_0.4_Mg_4_	20.3 ± 0.86 ab	17.3 ± 0.16 a	0.442 ± 0.025 b	0.231 ± 0.004 b	282 ± 5.44 a	235 ± 14.5 a	6.0 ± 0.42 b	7.7 ± 0.15 b
N_0.3_Mg_0_	13.9 ± 0.45 b	15.7 ± 0.26 b	0.291 ± 0.007 c	0.354 ± 0.005 a	275 ± 14.4 a	292 ± 3.67 a	6.9 ± 0.31 b	9.1 ± 0.49 a
N_0.3_Mg_2_	13.5 ± 0.73 b	14.5 ± 0.26 b	0.452 ± 0.012 b	0.248 ± 0.011 c	279 ± 11.9 a	268 ± 15.2 a	7.6 ± 0.27 ab	7.3 ± 0.06 b
N_0.3_Mg_4_	18.3 ± 0.40 a	17.8 ± 0.90 a	0.574 ± 0.002 a	0.286 ± 0.007 b	290 ± 5.72 a	265 ± 14.7 a	8.4 ± 0.26 a	8.4 ± 0.07 a
N_0.2_Mg_0_	15.7 ± 0.17 b	14.7 ± 0.32 b	0.315 ± 0.018 c	0.291 ± 0.001 b	258 ± 10.2 b	280 ± 11.8 a	6.5 ± 0.26 b	6.8 ± 0.24 b
N_0.2_Mg_2_	16.4 ± 0.03 ab	19.7 ± 0.41 a	0.415 ± 0.024 b	0.382 ± 0.014 a	274 ± 10.6 ab	286 ± 12.0 a	7.9 ± 0.41 a	8.6 ± 0.13 a
N_0.2_Mg_4_	17.8 ± 0.87 a	19.1 ± 0.68 a	0.502 ± 0.023 a	0.372 ± 0.003 a	286 ± 2.62 a	285 ± 4.78 a	9.0 ± 0.45 a	8.8 ± 0.11 a
Analysis of variance								
N	*	*	*	*	NS	*	NS	NS
Mg	*	*	*	NS	NS	NS	*	NS
N × Mg	*	*	*	*	NS	NS	*	*

Data are means ± SD (*n* = 3). Lowercase letters in the same column represent significant differences between different Mg concentrations for the same N treatment; * indicates significant differences, while NS indicates non-significant differences (*p* < 0.05).

### 3.3 Chlorophyll fluorescence parameters in leaves

**Figure 2:**
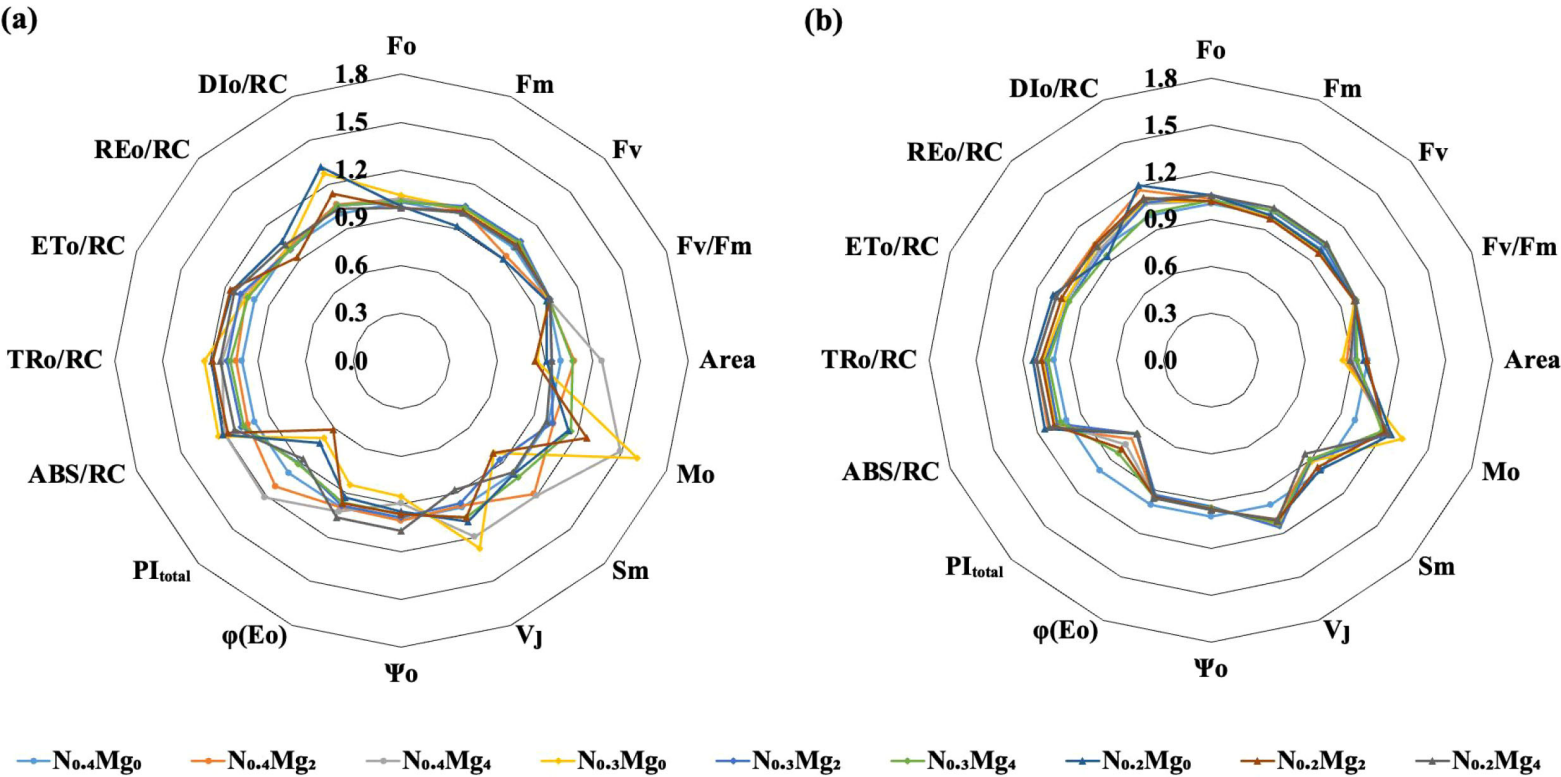
Chlorophyll fluorescence parameters in leaves under different treatments at the joining stage **(a)** and booting stage **(b)** of rice under different Mg and N treatments. Mg treatments—Mg_0_, Mg_2_, and Mg_4_ (corresponding to 0%, 2%, and 4% MgSO_4_•7H_2_O); N treatments—N_0.4_, N_0.3_, and N_0.2_ (corresponding to 0.4, 0.3, and 0.2 g N kg^−1^ soil). Fo—initial fluorescence intensity, Fm—maximal recorded fluorescence intensity, Fv—maximum variable fluorescence intensity, Area—PSII receptor library size, Mo—approximated initial slope of the fluorescence transient, Sm—normalized total complementary area, *V*_J_—relative variable fluorescence intensity at the J-step, Ψo—probability/efficiency (at *t* = 0) that a trapped exciton moves an electron into the electron transport chain beyond Q_A_, φEo—quantum yield of electron transport (at *t* = 0), and PI_total_—performance index (potential) for energy conservation from exciton to the reduction of PSI end acceptors. Data are means (*n* = 3).

Foliar Mg application significantly modulated chlorophyll fluorescence characteristics across different N application levels, with more pronounced effects observed at the jointing stage than at the booting stage (**Figure 2**; **Table 4**). Compared with N_0.4_, N_0.3_ and N_0.2_ treatments markedly increased Fo, Sm, ABS/RC, TRo/RC, ETo/RC, and DIo/RC. This indicates that reduced N supply impaired energy absorption and dissipation. In contrast, other key indicators of PSII photochemical performance, such as Fm, Area, φEo, Ψo, and PI_total_, declined under N_0.3_ and N_0.2_ treatments. Notably, these responses of fluorescence parameters to reduced N were substantially alleviated by Mg application.

**Table 4:** Two-factor analysis of chlorophyll fluorescence parameters at the jointing stage and booting stage of rice under different Mg and N treatments.

Analysis of variance	Jointing stage			Booting stage		
	N	Mg	N × Mg	N	Mg	N × Mg
Fo	*	NS	NS	NS	NS	NS
Fm	*	*	NS	NS	NS	NS
Fv	NS	NS	NS	NS	NS	NS
**Fv/Fm**	**NS**	*	*	NS	NS	NS
ABS/RC	*	NS	*	NS	NS	NS
TRo/RC	*	NS	NS	*	NS	NS
ETo/RC	*	NS	NS	NS	NS	NS
REo/RC	NS	NS	NS	NS	NS	NS
DIo/RC	*	*	*	NS	NS	NS
**Area**	*	*	**NS**	NS	NS	*
Mo	NS	*	*	NS	NS	*
Sm	*	*	*	NS	*	NS
*V*_J_	NS	*	*	NS	NS	NS
Ψo	*	NS	*	NS	NS	NS
φ(Eo)	*	*	NS	NS	NS	NS
**PI_total_**	*	*	*	*	NS	*

* indicates significant differences, while NS indicates non-significant differences (*p* < 0.05).

PI_total_ is one of the most important and sensitive parameters for evaluating PSII efficiency. Mg application consistently improved PI_total_ across all N treatments at the jointing stage. Compared with Mg_0_, Mg_4_ increased PI_total_ by 21.3%, 33.8%, and 19.6% under N_0.4_, N_0.3_, and N_0.2_, respectively. Similarly, Mg_2_ enhanced PI_total_ by 11.7% and 32.2% under N_0.4_ and N_0.3_, respectively, but caused a decline under N_0.2_. At the booting stage, the effects of Mg on PI_total_ varied with N level. Under high N supply (N_0.4_), Mg application resulted in a reduction in PI_total_, whereas under moderate and low N conditions, Mg supplementation significantly increased P_Itotal_ by 25.4% (N_0.3_Mg_4_) and 20.4% (N_0.2_Mg_2_) compared with the respective Mg_0_ treatments.

### 3.4 OJIP curves of chlorophyll fluorescence

**Figure 3:**
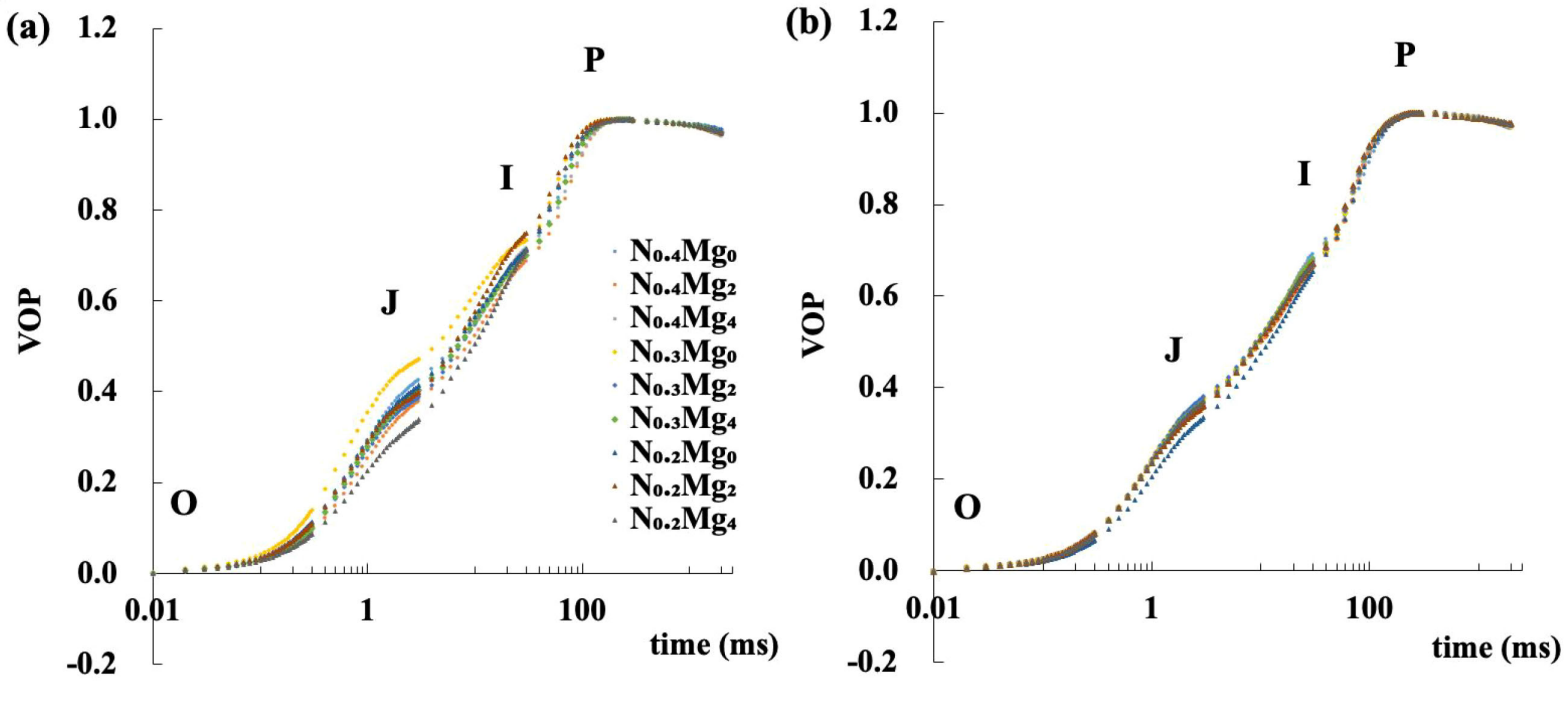
Relative variable fluorescence of functional leaves at the jointing stage **(a)** and booting stage **(b)** of rice under different Mg and N treatments. Mg treatments—Mg_0_, Mg_2_, and Mg_4_ (corresponding to 0%, 2%, and 4% MgSO_4_•7H_2_O); N treatments—N_0.4_, N_0.3_, and N_0.2_ (corresponding to 0.4, 0.3, and 0.2 g N kg^−1^ soil). The data corresponding to each point in the graph were expressed as relative values normalized to the N_0.4_Mg_0_ treatment. VOP = (Ft − Fo)/Fv; Ft—fluorescence intensity for corresponding time, Fo—initial fluorescence intensity, Fv—maximum variable fluorescence intensity, and VOP—relatively variable fluorescence intensity. The O–J–I–P phases represent the stepwise reduction of PSII electron acceptors during fluorescence induction. Data are means (*n* = 3).

**Figure 4:**
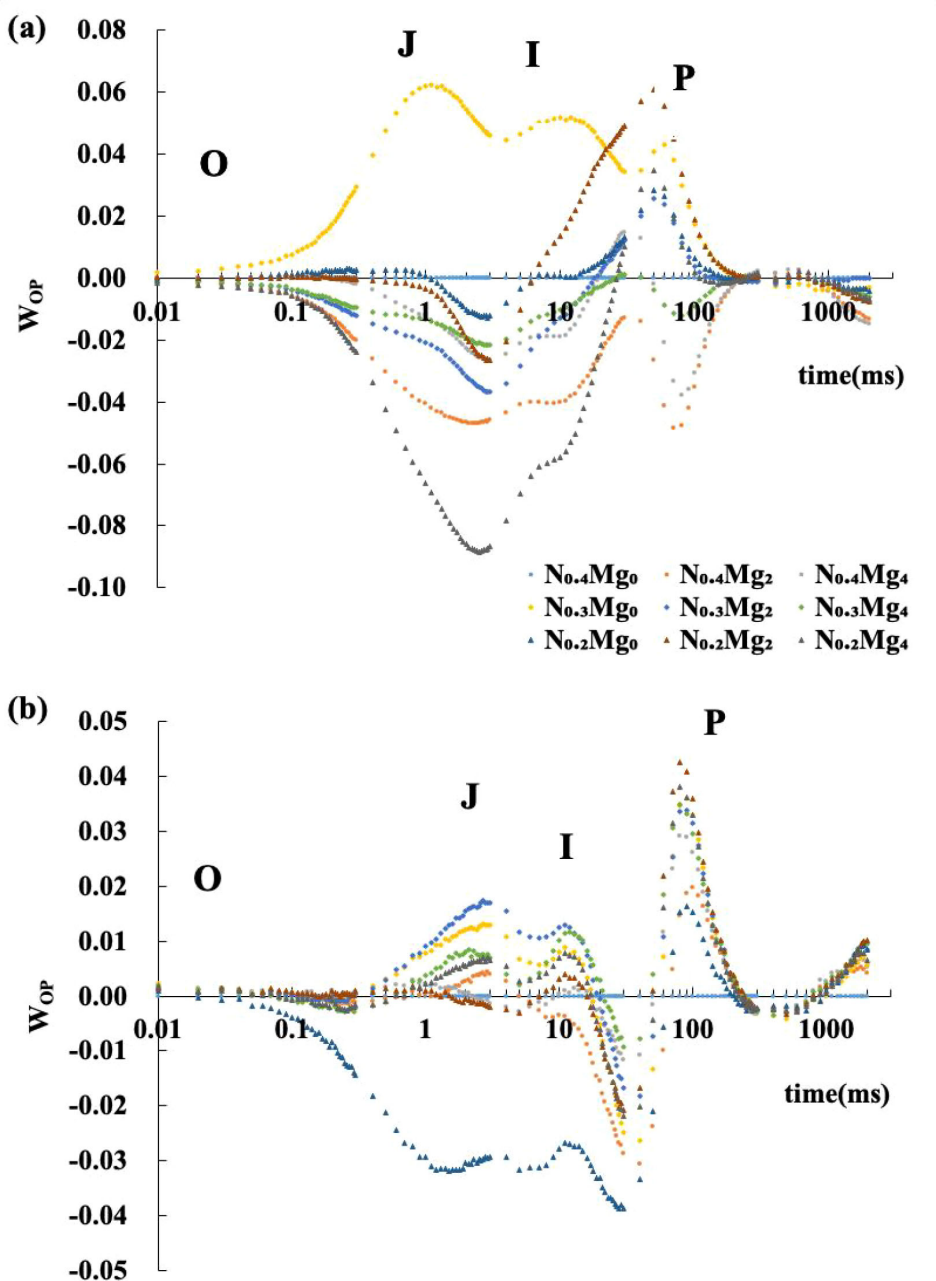
Kinetic difference of W_OP_ of functional leaves at the jointing stage **(a)** and booting stage **(b)** under different Mg and N treatments. Mg treatments—Mg_0_, Mg_2_, and Mg_4_ (corresponding to 0%, 2%, and 4% MgSO_4_•7H_2_O); N treatments—N_0.4_, N_0.3_, and N_0.2_ (corresponding to 0.4, 0.3, and 0.2 g N kg^−1^ soil). The data corresponding to each point in the graph were expressed as relative values normalized to the N_0.4_Mg_0_ treatment. Data are means of three biological replicates (*n* = 3). WOP = VOP (treatment) − VOP (N_0.4_Mg_0_) The O–J–I–P phases represent the stepwise reduction of PSII electron acceptors during fluorescence induction. Data are means (*n* = 3).

OJIP fluorescence curves showed an overall increase when N fertilizer was reduced by 25%, whereas foliar Mg application generally led to a decline (**Figures 3**, **4**). These changes were more pronounced at the jointing stage than at the booting stage. At the jointing stage, the overall fluorescence intensity, including J-, I-, and P-phases, decreased significantly with Mg_2_ and Mg_4_ compared with Mg_0_. The most pronounced decline occurred under N_0.2_Mg_4_, followed by N_0.4_Mg_2_, whereas N_0.3_Mg_0_ showed a marked increase. At the booting stage, a significant decrease in the OJIP curve was observed only under N_0.2_Mg_0_. The J- and I-phases decreased under N_0.4_Mg_4_ and N_0.3_Mg_4_ compared with their Mg_0_ counterparts, but increased significantly under N_0.2_Mg_4_ compared with N_0.2_Mg_0_.

### 3.5 Soluble sugar contents in leaves

**Figure 5:**
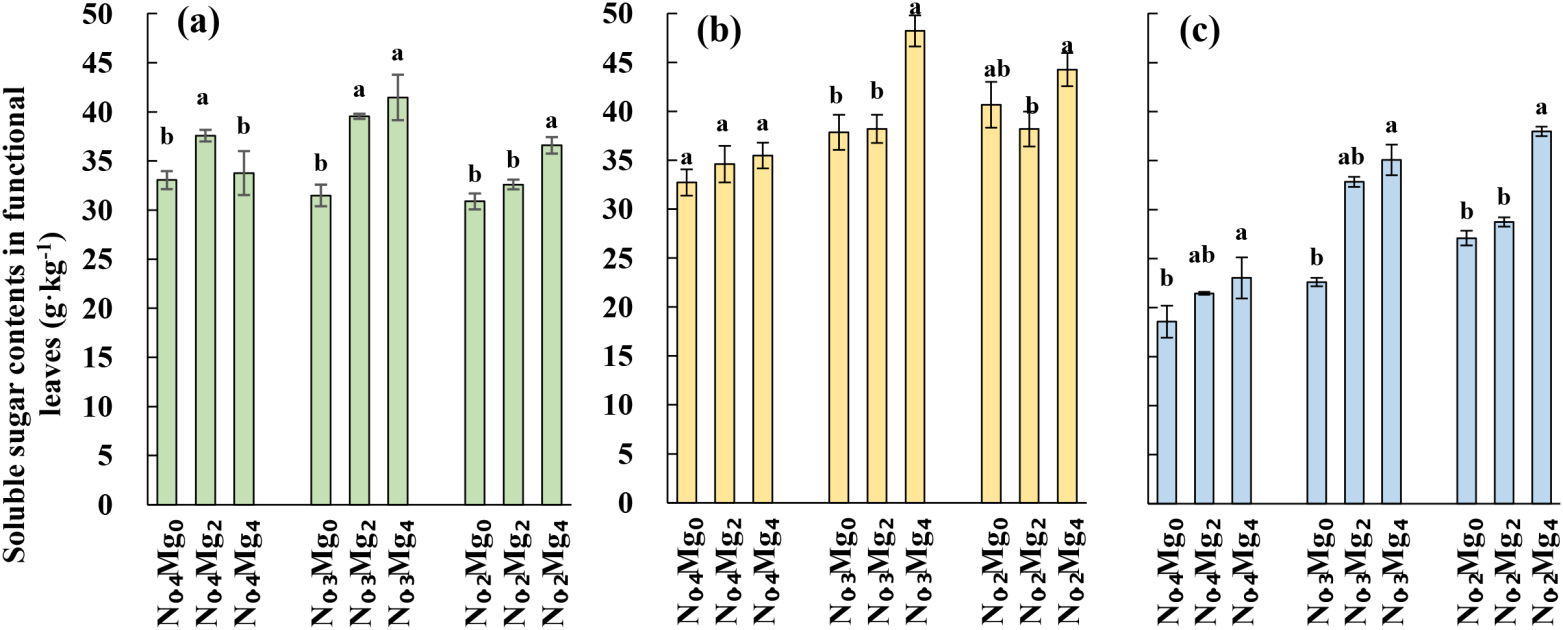
Soluble sugar in functional leaves of rice under different Mg and N treatments. Mg treatments—Mg_0_, Mg_2_, and Mg_4_ (corresponding to 0%, 2%, and 4% MgSO_4_•7H_2_O); N treatments—N_0.4_, N_0.3_, and N_0.2_ (corresponding to 0.4, 0.3, and 0.2 g N kg^−1^ soil). Soluble sugar contents in functional leaves **(a)** at the jointing stage, **(b)** at the booting stage, and **(c)** at the harvesting stage.

N_0.3_ and N_0.2_ treatments significantly decreased soluble sugar content at the jointing, booting, and harvest stages (**Figure 5**). Across all N levels, foliar Mg application consistently increased soluble sugar accumulation, with the strongest effect observed under the Mg_4_ treatment. At the jointing stage, soluble sugar content reached a maximum of 41.5 g kg^−1^ under N_0.3_Mg_4_, representing a 31.7% increase compared with N_0.3_Mg_0_, while N_0.2_Mg_4_ increased soluble sugar content by 18.5% compared to N_0.2_Mg_0_. At the booting stage, Mg application enhanced soluble sugar contents by 27.4% and 8.8% under Mg_2_ and Mg_4_, respectively, across both N_0.2_ and N_0.3_ levels. At harvest, soluble sugar content increased by 55% and 40.3% under Mg_2_ and Mg_4_, respectively. Although soluble sugar content generally declined under high N supply (N_0.4_), Mg application still resulted in a significant increase at harvest under the Mg_4_ treatment.

### 3.6 Starch and protein contents in grains

**Figure 6:**
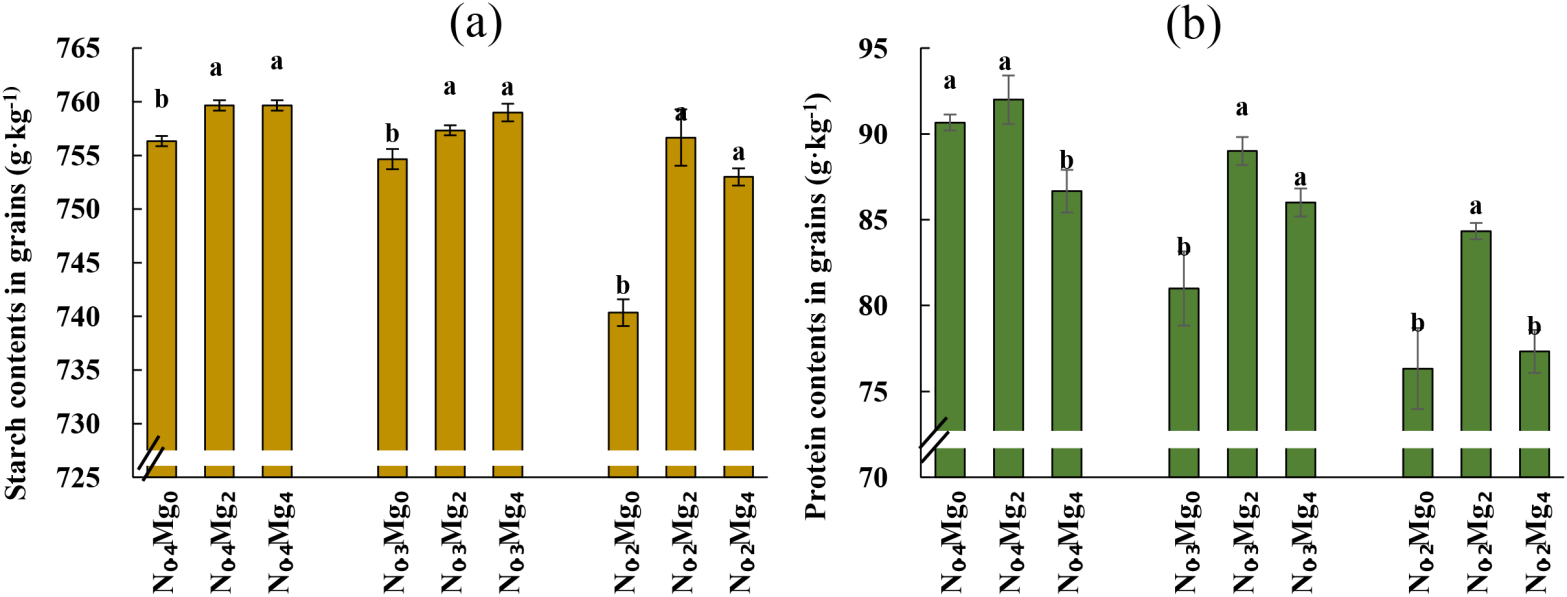
Starch and protein contents in grains of rice under different Mg and N treatments. Mg treatments—Mg_0_, Mg_2_, and Mg_4_ (corresponding to 0%, 2%, and 4% MgSO_4_•7H_2_O); N treatments—N_0.4_, N_0.3_, and N_0.2_ (corresponding to 0.4, 0.3, and 0.2 g N kg^−1^ soil). Starch **(a)** and protein **(b)** contents in grains. The bar letters represent significant differences between different Mg concentrations for the same N treatment. Data are means (*n* = 3).

Starch accumulation in grains was significantly affected by N and Mg application (**Figure 6a**). N_0.2_ and N_0.3_ treatments markedly decreased grain starch contents, whereas Mg application consistently promoted starch accumulation across different N levels. The highest starch contents (759.7 g kg^−1^) occurred under N_0.4_Mg_2_ and N_0.4_Mg_4_ treatments, while the lowest value (740.3 g kg^−1^) was observed under N_0.2_Mg_0_. Compared with Mg_0_, Mg_2_ and Mg_4_ increased starch contents by 0.44% and 0.45% under N_0.4_, by 0.35% and 0.57% under N_0.3_, and by 2.21% and 1.71% under N_0.2_, respectively. Thus, Mg supplementation promoted starch accumulation across a gradient of nitrogen supply, with more pronounced effects under reduced N levels.

Grain protein contents were significantly reduced under reduced N supply but increased after Mg application under the N_0.3_ and N_0.2_ conditions (**Figure 6b**). The highest protein contents (92.0 g kg^−1^) occurred under N_0.4_Mg_2_ treatment, whereas the lowest (76.3 g kg^−1^) was under N_0.2_Mg_0_ treatment. Compared with Mg_0_, Mg_2_ increased protein contents by 1.43%, whereas Mg_4_ decreased it by 4.45% under N_0.4_. Under N_0.3_, Mg_2_ and Mg_4_ increased protein contents by 9.88% and 6.17%, respectively, and under N_0.2_, by 10.53% and 1.35%, respectively. These findings demonstrate that Mg application enhances grain protein accumulation under variable N applications, with more pronounced effects under lower N supply.

### 3.7 Grains weight and components

Grain dry weight reduced with N fertilizer decrease, while foliar Mg supplementation consistently enhanced grain biomass across a gradient of N supply. The highest grain weight (151 g pot^−1^) was recorded under N_0.4_Mg_4_, which was 11.7% higher than N_0.4_Mg_0_. Under N_0.3_, Mg_2_ and Mg_4_ increased grain weight by 17.0% and 21.9%, respectively. Under N_0.2_, there was no difference between Mg_2_ and Mg_0_, while Mg_4_ decreased grain weight by 9.7% compared with Mg_0_ (**Table 5**).

**Table 5:** Grain dry weights and components of rice under different Mg and N treatments.

Treatment	Dry weight of grain (g pot^−1^)	Effective tiller number	1,000-Grain weight (g)	Grain-filling percentage (%)
N_0.4_Mg_0_	135 ± 3.29 b	33.0 ± 0.82 b	22.2 ± 0.51 a	89.8 ± 3.72 a
N_0.4_Mg_2_	141 ± 0.80 b	37.5 ± 2.86 ab	21.3 ± 0.33 a	88.1 ± 1.61 a
N_0.4_Mg_4_	151 ± 1.29 a	43.0 ± 3.74 a	22.4 ± 0.81 a	90.6 ± 0.58 a
N_0.3_Mg_0_	98 ± 2.40 b	28.3 ± 2.87 b	22.8 ± 0.98 a	88.5 ± 0.63 a
N_0.3_Mg_2_	115 ± 4.17 a	34.5 ± 0.41 a	23.1 ± 0.40 a	89.3 ± 2.50 a
N_0.3_Mg_4_	119 ± 0.49 a	35.5 ± 0.41 a	22.3 ± 0.43 a	87.6 ± 1.22 a
N_0.2_Mg_0_	69 ± 0.05 c	24.5 ± 1.22 a	20.6 ± 1.22 b	79.5 ± 2.65 b
N_0.2_Mg_2_	84 ± 0.63 a	22.5 ± 0.41 a	24.0 ± 0.32 a	87.4 ± 2.21 a
N_0.2_Mg_4_	76 ± 0.70 b	22.0 ± 1.63 a	22.7 ± 0.29 ab	90.9 ± 1.82 a
Analysis of variance				
N	*	*	NS	*
Mg	*	*	NS	*
N × Mg	*	*	*	*

Data are means ± SD (*n* = 3). Lowercase letters in the same column represent significant differences between different Mg concentrations for the same N treatment; * indicates significant differences, while NS indicates non-significant differences (*p* < 0.05).

Effective tiller number, 1,000-grain weight, and grain-filling percentage decreased with N reduction. Mg application significantly increased the effective tiller number under N_0.4_ and N_0.3_ treatments, and increased the 1,000-grain weight and grain-filling percentage only at N_0.2_ treatment. N_0.4_Mg_4_ recorded the highest effective tiller number (43), while the highest 1,000-grain weight and grain-filling percentage occurred at N_0.2_Mg_2_ and N_0.2_Mg_4_, respectively.

## 4 Discussion

### 4.1 Enhancements of Mg uptake and photosynthetic framework through foliar Mg application

Both Mg and N are essential components of chlorophyll (Ceppi et al., 2012; Chen et al., 2019; Dölger et al., 2024; Hu et al., 2024). In this study, their interaction significantly influenced net photosynthetic rate, stomatal conductance, and transpiration efficiency (**Table 3**). Net photosynthetic rate, stomatal conductance, and transpiration rate tended to decline under relatively lower nitrogen application, while foliar Mg application improved these parameters. This variation was consistent with changes in leaf Mg contents under different N applications (**Figure 1**). Thus, foliar Mg application promotes Mg accumulation in leaves across a gradient of nitrogen supply, thereby maintaining nutrient homeostasis under variable N conditions. These results were also consistent with findings in citrus (Tang et al., 2012), *Arabidopsis* (Ogura et al., 2020), and maize (Jezek et al., 2015). In addition, previous studies showed that sufficient Mg supply in leaves and pods of beans doubled net CO_2_ assimilation and stomatal conductance compared with Mg-deficient plants (Geng et al., 2021). Likewise, Mg deficiency lowered RuBP carboxylase activity and transcript levels of its subunits in spinach, thereby restricting CO_2_ fixation (Ze et al., 2009). In this study, we did not focus on enzyme activity, but rather concentrated on the photosynthetic electron transfer by analyzing chlorophyll fluorescence parameters.

### 4.2 Photosynthetic enhancements obtained through betterments in energy transfer efficiency

The first step in photosynthesis is to obtain light energy through antenna complexes, followed by light energy transfer to PSII reaction centers where photochemical processes occur (Ouled Youssef and Krouma, 2021). Our results showed that N reduction decreased Fm, Area, φEo, Ψo, and PI_total_, while it increased Fo, Sm, ABS/RC, TRo/RC, ETo/RC, and DIo/RC (**Figure 2**, **Table 4**). Most of these effects were largely reversed by foliar Mg application. For example, Mg decreased DIo/RC, while it increased Area, φEo, and PI_total_. Specific energy fluxes per active PSII reaction center (ABS/RC, TRo/RC, ETo/RC, and DIo/RC) reflect energy allocation within PSII. Under stress conditions such as Fe imbalance or salt stress, ABS/RC increases because of inactivated PSII centers (Liu et al., 2020; Gao et al., 2022). After applying iron fertilizer, ABS/RC, TRo/RC, and DIo/RC decreased and ETo/RC and REo/RC increased, resulting in a more balanced distribution of energy (Gao et al., 2022). Similar to Fe, Mg can stabilize energy distribution in the photosynthetic electron transport chain and promote donor-side activity. As an important component of chlorophyll, Mg is involved in the absorption and distribution of light energy and ultimately PSII performance (Tränkner et al., 2018). PI_total_ is a comprehensive indicator of PSII performance (Kalaji et al., 2014). Its significant increase under Mg application across N treatments (**Figure 2**; **Table 4**) suggests that Mg enhances energy transfer efficiency in the photosynthetic electron transport chain, thereby supporting higher photosynthetic performance.

OJIP transients provide detailed insights into electron transport through PSII (Rapacz et al., 2019). In this study, variation in nitrogen application, foliar Mg spraying, and their interaction influenced the relative fluorescence intensity at the jointing stage, with most treatments showing lower values compared with the control, except for the N_0.3_Mg_0_ treatment. However, at the booting stage, this effect showed a reversed trend. Moreover, under relatively lower nitrogen levels, Mg application generally resulted in higher relative fluorescence intensity compared with treatments without Mg application. In particular, Mg had a more significantly positive effect on the fluorescence intensity at the N_0.2_ level (**Figures 3**, **4**). These results suggest that lower N application may induce photoinhibition, likely due to impaired electron transport activity (Mohotti and Lawlor, 2002; Chen et al., 2021), but that this effect is partly alleviated by foliar Mg application. The aforementioned fluorescence changes mainly occurred at the J, I, and P steps, suggesting potential constraints on the reoxidation of quinone A (QA) and on electron transport from plastoquinone (PQ) to cytochrome b6/f and PSI under relatively lower nitrogen availability. Some research also reported that environment stress, for example, excessive Fe, alters the electron transfer at the donor side of PSII, resulting in the further closure of the PSII reaction center with the electronic accumulation in quinone (QA and QB). The change of fluorescence at the I–P phase affects the electron transfer at the PSI (Liu et al., 2020).

### 4.3 Improvements in photosynthetic framework translated into increased carbohydrate

Carbohydrates are the primary products of photosynthesis, and they are translocated through the phloem to support plant growth and grain filling (Pask et al., 2012; Mu & Chen, 2021; Pask et al., 2011; Farhat et al., 2016). Mg not only improves photosynthesis by enhancing Mg contents in plants and facilitating energy transfer within PSII and PSI, but also plays a critical role in sucrose loading and transport (Cakmak and Kirkby, 2008; Karley and White, 2009). Supplementary Mg enhances sucrose distribution to sink organs and rapidly restores phloem transport in Mg-deficient plants (Peng et al., 2020). Our results showed that Mg supplementation enhanced soluble sugar accumulation in functional leaves (**Figure 5**) and promoted starch deposition in grains (**Figure 6**) under different nitrogen regimes, with responses varying depending on nitrogen availability. These results are consistent with citrus, where Mg fertilization boosts carbohydrate accumulation by enhancing Rubisco activity and chlorophyll content (Syvertsen et al., 2003; Tang et al., 2012). Moreover, previous research in rice also confirmed that foliar Mg promotes starch accumulation (Ali et al., 2021). Therefore, Mg fertilization can improve carbohydrate synthesis, transport, and partitioning, ultimately supporting higher grain filling and quality.

### 4.4 Enhancements in grain yield

Many studies have demonstrated that Mg fertilization can increase crop yield (Zhang et al., 2021; Ye et al.,2023; Wang et al., 2024; Wang et al., 2025; Zhao et al., 2025; Ding et al., 2006; Rodrigues et al., 2021; He et al., 2024). In the present study, foliar Mg application significantly enhanced grain weight of rice only at N_0.4_ and N_0.3_ levels, but not at the N_0.2_ level (**Table 5**). Under the N_0.4_ and N_0.3_ treatments, Mg application also increased the number of effective tillers, suggesting that the higher grain weight may be attributed to the greater number of effective tillers. Under the N_0.2_ treatment, although Mg application did not significantly increase overall grain weight, it enhanced 1,000-grain weight and grain-filling percentage. Previous studies have also shown that photosynthetic efficiency strongly influences pod and seed set in oilseed crops (Wang et al., 2016). In our experiment, foliar Mg supplementation stimulated CO_2_ assimilation and carbon partitioning, thereby enhancing photosynthetic capacity and promoting increases in effective tiller number and 1,000-grain weight across different nitrogen treatments.

## 5 Conclusion

Foliar Mg application significantly modulated photosynthetic performance and carbohydrate metabolism in rice across three nitrogen application levels. Mg treatment decreased DIo/RC and Fo while enhancing O–J and J–I fluorescence intensities, indicating reduced energy dissipation and improved photochemical efficiency of PSII. These changes promoted light energy utilization and photosynthetic capacity. Furthermore, Mg application enhanced the synthesis, transport, and accumulation of photosynthetic products, resulting in higher grain yield and quality. The yield increased by 4.42%–22.7%, and starch contents increased by 0.35%–2.21%. The beneficial effects of Mg were more evident under relatively lower nitrogen availability, suggesting that Mg supplementation helps maintain physiological performance under varying nitrogen conditions. In conclusion, this study demonstrates that the N_0.4_Mg_4_ treatment was more favorable for improving rice yield and quality, whereas the N_0.2_Mg_4_ treatment showed greater potential for enhancing NUE.

## Data Availability

The original contributions presented in the study are included in the article/supplementary material. Further inquiries can be directed to the corresponding authors.
