# Correction: Photosynthesis and yield enhancements in rice by foliar magnesium supply under variable soil nitrogen applications

**DOI:** 10.3389/fpls.2026.1909740

**Published:** 2026-06-24

**Authors:** Frontiers Production Office

**Affiliations:** Frontiers Media SA, Lausanne, Switzerland

**Keywords:** foliar spraying Mg fertilizer, N fertilizer, N and Mg interaction, photosynthesis, rice

Several sentences were replaced with only reference citations in error. These sentences have been restored alongside their citations. The added sentences are listed below:

“An adequate Mg fertilization rate can increase crop yield by an average of 8.5% worldwide and improve the quality of various agricultural products (Grzebisz, 2013; Hauer-Jakli & Trankner, 2019; Jaghdani et al., 2021; Andrzejewska et al., 2025; Wang et al., 2020; Chen et al., 2022)”.

“Nitrogen (N) is another essential nutrient and is fundamental to plant growth and agricultural production (Lee et al., 2020; Guo et al., 2021; Mu & Chen, 2021; You et al., 2023)”.

“There is an important interaction between Mg and N in various physiological and biochemical processes within plants (Lamichhane et al., 2023; Li et al., 2023; Tian et al., 2023; Yang et al., 2024)”.

“Both Mg and N are essential components of chlorophyll (Ceppi et al., 2012; Chen et al., 2019; Dölger et al., 2024; Hu et al., 2024)”.

“Carbohydrates are the primary products of photosynthesis, and they are translocated through the phloem to support plant growth and grain filling (Pask et al., 2012; Mu & Chen, 2021; Pask et al., 2011; Farhat et al., 2016)”.

“Many studies have demonstrated that Mg fertilization can increase crop yield (Zhang et al., 2021; Ye et al., 2023; Wang et al., 2024; Wang et al., 2025; Zhao et al., 2025; Ding et al., 2006; Rodrigues et al., 2021; He et al., 2024)”.

The original version of this article has been updated.

